# The Reproductive Autonomy Scale: German validation and application in an Austrian student population

**DOI:** 10.3389/fpubh.2025.1712168

**Published:** 2026-01-14

**Authors:** Bettina Böttcher, Magdalena Flatscher-Thoeni, Elisabeth Reiser, Bettina Toth, David Riedl

**Affiliations:** 1 Department of Gynecological Endocrinology and Reproductive Medicine, Medical University of Innsbruck, Innsbruck, Austria; 2 Department of Public Health, Health Services Research and Health Technology Assessment, UMIT TIROL – University for Health Sciences and Technology, Hall in Tirol, Austria; 3 Department of Psychiatry, Psychotherapy, Psychosomatics and Medical Psychology, University Hospital of Psychiatry II, Medical University of Innsbruck, Innsbruck, Austria; 4 Ludwig Boltzmann Institute for Rehabilitation Research, Vienna, Austria

**Keywords:** contraception, gender differences, reproductive autonomy, reproductive health, shared decision- making

## Abstract

**Introduction:**

Reproductive autonomy is defined as the right to make independent decisions regarding sexuality, contraception, pregnancy, and childbirth without coercion. The Reproductive Autonomy Scale (RAS) is the first instrument to assess decision-making, freedom from coercion, and communication in reproductive contexts and has been validated in several languages. The objective of this study was to validate the RAS in a German context and explore its application in an Austrian student population, including its use with men.

**Materials and methods:**

A comprehensive set of descriptive statistics was derived for the cohort (*n* = 625). The psychometric properties of the RAS were evaluated using internal consistency (Cronbach’s *α*) and corrected item-total correlations. Measurement invariance across gender was assessed explored using multi-group confirmatory factor analysis (CFA). Gender-specific item response patterns were analyzed, and associations with sociodemographic variables were examined using univariate ANOVAs and t-tests with Bonferroni correction.

**Results:**

Psychometric analysis demonstrated satisfactory item-total correlations and factor loadings, supporting the three-factor structure of the RAS. Internal consistency of the subscales for decision-making and freedom from coercion was satisfactory, while the communication subscale showed marginally lower reliability (*α* = 0.67). CFA confirmed the proposed model. Configural invariance was not supported, and exploratory comparisons of factor loadings indicated gender differences. Female participants reported higher rates of sole decision-making regarding reproductive autonomy. For ‘freedom from coercion’ and ‘communication’, no substantial gender differences were found.

**Conclusion:**

The RAS is a reliable tool for analysing reproductive autonomy in German-speaking populations. However, its use in men and diverse individuals is constrained, necessitating adaptations. Austrian students showed high reproductive autonomy, influenced by age and sexual orientation in one subscale. Yet, women continue to bear greater responsibility for contraception, highlighting the need for better sexual education and shared responsibility between partners.

## 1 Introduction

Reproductive autonomy constitutes a critical subdomain of personal autonomy (1) with significant implications for clinical care, public health, and health equity. Reproductive autonomy refers to the right and capacity of individuals to make informed and voluntary decisions about their reproductive health—including the use of contraception, the timing and number of pregnancies, and the decision to continue or terminate a pregnancy—free from coercion, external interference, or structural barriers (2). As such, reproductive autonomy functions both as a core principle of biomedical ethics—reflecting respect for patient autonomy—and as a legally codified human right, recognized by international health and human rights frameworks (3).

Reproductive autonomy is a multidimensional concept operating across several interrelated levels, each of which is directly relevant to medical and public health practice. At the individual level, for example, it encompasses a person’s knowledge, attitudes, beliefs, age, cognitive and emotional capacities, and health literacy, all of which influence their ability to make informed reproductive decisions. At the relational level, reproductive autonomy is shaped by interpersonal dynamics, particularly within intimate partnerships, families and social networks. Here, power imbalances or support structures can significantly affect one’s decision-making capacity. At the community and societal levels, broader structural determinants, including access to healthcare, education, employment, and prevailing cultural or gender norms, either facilitate or constrain reproductive autonomy. These factors highlight that reproductive autonomy is not just an individual matter, but one embedded in relational, institutional and systemic contexts that must be considered in clinical care and policy (4).

Given its multidimensional nature and its centrality to reproductive health, gender equity, and human rights, reproductive autonomy represents a critical area for empirical inquiry. However, systematic research has been hindered by the lack of standardized, psychometrically validated instruments capable of capturing the complexity of this construct across diverse populations and contexts. To address this gap, Upadhyay et al. (5) developed the Reproductive Autonomy Scale (RAS)—a theoretically grounded and empirically validated measure. The RAS enables the quantification of reproductive autonomy, particularly in relation to contraceptive and pregnancy-related decision-making.

The present study aims to advance reproductive autonomy research by addressing key gaps in measurement and inclusivity. Specifically, we aim to: Translate and psychometrically validate the RAS for use in the German language; Evaluate its applicability across genders, including men and diverse individuals and explore preliminary patterns of reproductive autonomy within a sample of Austrian university students. By extending the scope of the RAS beyond its original cultural and demographic context, this study seeks to enable more equitable, inclusive and empirically grounded assessments of reproductive autonomy in diverse populations.

## 2 Methods

### 2.1 Procedure

The study was conducted online with SurveyMonkey© in Austria among university students across multiple academic disciplines in October 2023. The ethical committee of the Medical University of Innsbruck, Austria, approved the study (AN 1012/2022). It was conducted in accordance with the Declaration of Helsinki. Participation in the study was voluntary, and data was collected anonymously. The invitation to participate in the study including web links and QR codes to the questionnaires, was distributed online via emails to various study program directors and study representatives or school principals. Consent to participate in the study was given online at the beginning of the questionnaire.

Data processing was done in accordance with the EU General Data Protection Regulation (GDPR).

Inclusion criteria were sufficient German language skills and current registration as a student in Tyrol. Participation was anonymous and voluntary.

### 2.2 Questionnaire and its validation

The translation of the RAS from English into German was carried out according to established scientific standards for linguistic validation. The original version of Upadhyay et al. (5) in English is available online. In addition to a forward translation and backward translation, the questionnaire was tested for comprehensibility in a small sample of *n* = 10 before the final version was used. When translating the questionnaire, gender-appropriate language was deliberately used to make the content equally accessible and inclusive for people of all gender identities. Bilingual experts as well as professionals in reproductive health approved the final version.

The questionnaire comprises 14 items organized into three subscales that capture distinct dimensions of reproductive autonomy: Freedom from Coercion, Communication, and Decision-Making.

The Freedom from Coercion subscale assesses the extent to which individuals experience coercive behaviors from their partners in matters related to contraception and pregnancy. It includes items that reflect pressure, manipulation, or control over reproductive decisions.

The Communication subscale evaluates the degree and quality of communication between partners about reproductive topics, such as contraception use, sexual activity, and pregnancy intentions. It reflects the respondent’s ability to discuss these issues openly within the relationship.

The Decision-Making subscale examines who holds authority over reproductive decisions in the relationship. It captures the dynamics of individual versus shared control over choices related to reproduction.

Each item within the Freedom from Coercion and Communication subscales is rated on a four-point Likert scale, with response options reflecting increasing levels of agreement or frequency. These responses are scored from 0 to 3 points, allowing for differentiation in the degree of perceived coercion or quality of communication. In contrast, the Decision-Making subscale uses a three-option response format to indicate who holds decision-making authority—whether the respondent, their partner, or both jointly. Responses in this subscale are scored from 0 to 2 points, capturing the extent of individual or shared agency in reproductive decisions.

### 2.3 Statistics

Descriptive statistics are presented for the whole sample. We only included participants who completed key sociodemographic data and the RAS. Due to the low number of gender diverse individuals all subsequent analyses were only conducted for male and female participants. All psychometric analyses were conducted using the full original Likert response scales. For the gender-specific descriptive item analysis, response categories were collapsed into three groups (strongly disagree/disagree/agree–strongly agree) exclusively for visualization and interpretive clarity. This recoding did not influence any reliability, CFA, or inferential analyses.

In a first step, psychometric properties of the RAS were evaluated. Reliability was tested by calculation of the internal consistency (Cronbachs’ Alpha *α*) of each scale. As a rule of thumb, Cronbach’s *α* > 0.90 is considered excellent, while *α* > 0.80 is considered good, *α* > 0.70 acceptable, *α* > 0.60 questionable and *α* < 0.60 poor (6). Additionally, corrected item-total correlation was calculated with values of *r* > 0.3 considered to be acceptable. Confirmatory factor analysis (CFA) was performed to validate the previously proposed three factor solution for the RAS. Patterns of missing data was analyzed for items of each subscale separately with Littles MCAR test and data was imputed using the Expectation–Maximization-Algorithm if data was completely missing at random (MCAR). In case of non MCAR data were excluded. To determine the factor solution’s goodness of fit, Pearson’s chi-squared test (χ^2^), degree of freedom (df), Tucker-Lewis index (TLI), comparative fit index (CFI) and root mean square error of approximation (RMSEA) with lower and higher bounds of the 95% confidence interval (CI) were calculated. Based on modification indices additional paths between error-terms were added to enhance the fit of the model. In order to maintain parsimony, modification indices were considered only when a substantive basis was present, supported by empirical considerations. To evaluate measurement invariance in terms of gender, we tested for configural, metric and scalar invariance between the two groups (male vs. female). Configural invariance tests whether the same factor structure holds across groups, metric invariance tests whether factor loadings are equivalent, and scalar invariance tests whether item intercepts are equivalent. In the context of the RAS, these indicate whether the scale measures reproductive autonomy in the same way for male and female participants. Configural invariance was tested by running separate CFAs for each group to ensure that the factor structure remained consistent across the groups. After establishing configural invariance, we proceeded to test for metric invariance. This involved constraining factor loadings across groups and comparing the fit of the constrained model to the unconstrained model. Following metric invariance, scalar invariance was examined by constraining both factor loadings and intercepts of observed variables across groups. Model fit was assessed to determine equivalence of intercepts. If the assumption of measurement invariance was not met, pairwise parameter comparison will be conducted to identify critical ratios between individual unconstrained parameters (i.e., values below −1.96 and above 1.96 indicating a significant difference). Throughout the analysis, we rigorously evaluated model fit using established indices, including the Comparative Fit Index (CFI), Tucker-Lewis Index (TLI), and Root Mean Square Error of Approximation (RMSEA), ensuring the validity of comparisons. Acceptable goodness of fit was defined as RMSEA values of <0.06–0.08 and CFI/TLI values >0.90 (6, 7). Factor loadings >0.40 were considered acceptable (8).

In a last step, a gender-specific analysis of the individual item responses was conducted. To facilitate interpretability, the participants scorings were reduced to three answer categories (strongly disagree vs. disagree vs. agree / strongly agree). To test the associations of other sociodemographic variables univariate analyses of variance and Pearson t-tests were calculated. For multiple comparisons, Bonferroni-corrected *p*-values were calculated to avoid alpha error inflation. All analyses were conducted with IBM SPSS (v21) and IBM SPSS AMOS (v29). *p*-values below 0.05 were considered statistically significant.

## 3 Results

### 3.1 Study population—descriptive statistics

The target population for this study was university students enrolled in academic institutions in the Tyrol region. The study population comprised all individuals who received the online survey link via program directors and student representatives. Of 939 students who accessed the survey, 625 participants who completed key sociodemographic variables and the full RAS constituted the final analytic sample. In German, the term “Geschlecht” refers to both sex (the biological aspect) and gender (the social and cultural aspect). Most respondents were female (83.2%), of Austrian origin (60.6%), and heterosexual (81.3%) (Table 1).

**Table 1:** Sociodemographic data.

Gender		
Female	520	83.2%
Male	100	16.0%
Diverse	5	0.8%
Nationality		
Austrian	379	60.6%
German	142	22.7%
Italian	86	13.8%
Other	18	2.9%
Religious beliefs		
Atheism	113	18.1%
Christian	454	72.6%
Buddhism	3	0.5%
Islam	4	0.6%
None/other	51	8.2%
Study subject		
Social sciences	77	12.3%
Natural sciences	148	23.7%
Health sciences	400	64.0%
Highest education parents		
School not finished	3	0.4%
Compulsory school	91	12.6%
Apprenticeship	189	26.3%
High school diploma	151	21.0%
University	281	39.0%
Missing	5	0.7%
Sexuality		
Heterosexual	585	81.3%
Homosexual/ Bisexual	109	15.1%
Another identity	26	3.6%

### 3.2 Psychometric and factorial validity of the RAS

The psychometric properties of the RAS items are presented in Table 2. Generally, item-total correlation was acceptable for all items (*r* = 0.32–0.61), except item 7 which was borderline acceptable (*r* = 0.30). Factor loadings were within the expected range for all items, except for item 14, which showed a lower-than-expected loading (*β* = 0.36). Item 14 (‘*If I really did not want to become pregnant, I could get my partner to agree with me*’) embeds a pregnancy-specific negotiation scenario. While this captures reproductive autonomy for women, it may be difficult for male participants to interpret literally and may instead reflect more general relational influence. This conceptual mismatch potentially explains why the item aligned weakly with the communication factor. The internal consistency for the ‘decision-making’ subscale (*α* = 0.75) and the ‘freedom from coercion subscale’ (*α* = 0.70) were acceptable, while the ‘communication’ subscale scored slightly below the acceptable range (*α* = 0.67). Computed item-scale statistics indicated that deleting item 14 would result in a slightly better internal consistency of the subscale (*α* = 0.69).

**Table 2:** Psychometric properties of the items and scales of the reproductive autonomy scale (RAS).

Nr	Item	Mean	SD	% miss	Item-total correlation	Factor loading	Cronbach alpha
1. Decision-making (range: 1–3)		2.42	0.46	6.9%			0.75
1.	Who has the most say about whether you use a method to prevent pregnancy?	2.35	0.55	7.2%	0.51	0.60	
2.	Who has the most say about which method you would use to prevent pregnancy?	2.58	0.59	7.2%	0.61	0.74	
3.	Who has the most say about when you have a baby in your life?	2.28	0.56	7.4%	0.54	0.60	
4.	If you became pregnant but it was unplanned, who would have the most say about whether you would raise the child, seek adoptive parents. or have an abortion?	2.49	0.70	7.5%	0.56	0.67	
2. Freedom from coercion (range: 1–4)		1.09	0.26	9.7%			0.70
5.	My partner has stopped me from using a method to prevent pregnancy when I wanted to use one.	3.89	0.40	9.7%	0.53	0.44	
6.	My partner has messed with or made it difficult to use a method to prevent pregnancy when I wanted to use one.	3.85	0.46	9.9%	0.56	0.50	
7.	My partner has made me use a method to prevent pregnancy when I did not want to use one.	3.93	0.30	10.1%	0.30	0.44	
8.	If I wanted to use a method to prevent pregnancy my partner would stop me.	3.94	0.30	10.0%	0.51	0.78	
9.	My partner has pressured me to become pregnant.	3.97	0.21	10.0%	0.44	0.61	
3. Communication (range: 1–4)		3.60	0.41	10.1%			0.67
10.	My partner would support me if I wanted to use a method to prevent pregnancy	3.85	0.43	10.4%	0.39	0.51	
11.	It is easy to talk about sex with my partner.	3.52	0.67	10.6%	0.50	0.64	
12.	If I did not want to have sex I could tell my partner.	3.64	0.53	10.3%	0.53	0.68	
13.	If I was worried about being pregnant or not being pregnant I could talk to my partner about it.	3.63	0.64	11.5%	0.52	0.63	
14.	If I really did not want to become pregnant, I could get my partner to agree with me.	3.38	0.76	11.8%	0.32	0.36	

### 3.3 Factor structure of the RAS

To test the factorial validity of the RAS a CFA was conducted based on the previously proposed three factor structure of the scale. In the total sample a good model fit was observed (χ^2^ = 186,324, *p* < 0.001; CMIN/DF = 2.552; TLI = 0.923, CFI = 0.938; RMSEA = 0.050, 95%: 0.041–0.059).

For sensitivity reasons, we tested the factorial validity of the 3-factor model without item 7 and without item 14, individually and combined. The exclusion of item 7 did not increase the internal consistency of the factor ‘freedom from coercion’ and the overall model fit decreased. The exclusion of item 14 lead to a slightly improved internal consistency of factor 3 (*α* = 0.69) but no superior model fit was observed. Thus, we concluded to continue analyses with the initially proposed 3-factor solution. An overview of the models fit indices is shown in Table 3.

**Table 3:** Fit indices for the different models of confirmatory factor analysis (CFA).

	*n*	χ^2^	*p*	CMIN/DF	TLI	CFI	RMSEA	95% CI	PCLOSE
Total sample	625	186.324	<0.001	2.552	0.923	0.938	0.050	0.041–0.059	0.49
Total sample (without item 7)	625	179.498	<0.001	2.943	0.913	0.932	0.056	0.046–0.065	0.15
Total sample (without item 14)	625	166.515	<0.001	2.730	0.924	0.940	0.053	0.043–0.062	0.31
Total sample (without item 7 and 14)	625	159.971	<0.001	3.199	0.914	0.935	0.059	0.049–0.070	0.06
Females	525	156.654	<0.001	2.146	0.921	0.937	0.047	0.037–0.057	0.69
Males	100	97.409	0.030	1.334	0.867	0.893	0.058	0.037–0.057	0.32
Males (without item 10)	100	63.670	0.383	1.044	0.983	0.987	0.021	0.000–0.065	0.82

### 3.4 Assessment of measurement invariance

To evaluate measurement invariance the model was separately tested for male and female participants in a first step. A good fit was observed in the female and all paths remained statistically significant (all *p* < 0.001). Details on factor loadings can be requested from the corresponding author. In the male sample on the other hand, model fit was substantially worse and the path from factor 3 with item 10 (‘My partner would support me if I wanted to use a method to prevent pregnancy’) was not statistically significant (*p* = 0.10). Exclusion of the item lead to a substantially improved and good model fit in the male sample. The item measures not only communication but also the partner’s stance toward contraceptive responsibility, which may vary depending on gender and relationship dynamics. For male respondents, this item may index their perception of their partner’s autonomy rather than their own, which reduces coherence with the communication construct. For an overview of model fit indices of the tested models refer to Table 3.

Based on the results of the CFAs, the assumption of configural invariance was not supported because model fit for men was substantially poorer (CFI = 0.893, TLI = 0.867, RMSEA = 0.058) compared to women (CFI = 0.937, TLI = 0.921, RMSEA = 0.047), and one path (factor 3 → item 10) was nonsignificant. We proceeded with exploratory metric testing to better understand sources of group difference, acknowledging that this is not a formal invariance conclusion. When comparing the measurement weights of male and female participants invariance testing indicated significant differences between the groups (χ^2^ = 40.032, *p* < 0.001). A pairwise comparison of the unconstrained parameters for the factor loadings indicted a significant gender difference for the path of factor 2 and item 7 (*z* = 2.108) as well as factor 3 and item 10 (*z* = 3.337).

### 3.5 Gender-specific analyses of the RAS subscales

**Figure 1:**
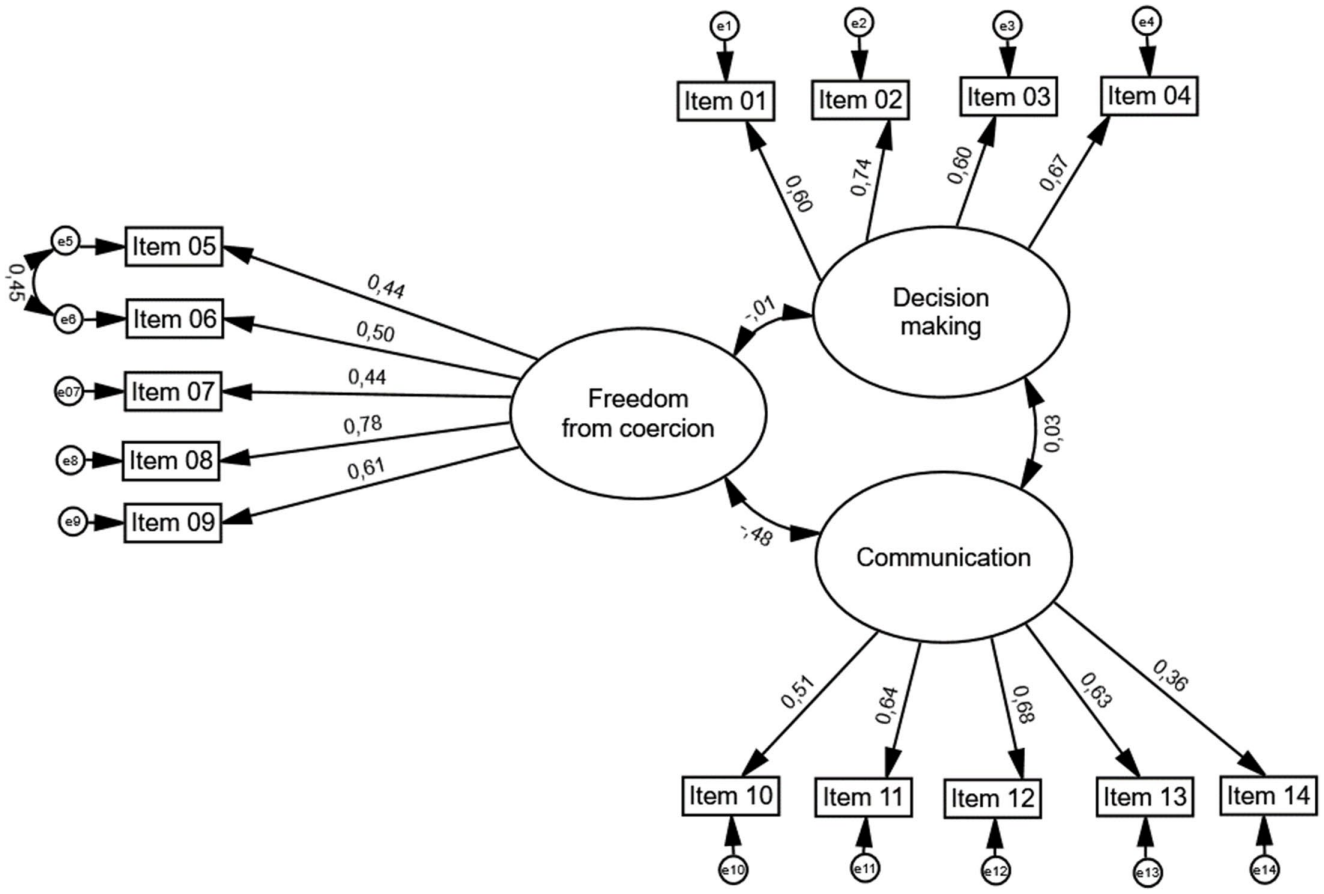
Confirmatory factor analysis (CFA) for the proposed factor structure of the Reproductive Autonomy Scale (RAS). Ellipsoids represent domains, rectangles represent RAS items and their item number, and circles represent error terms. Numeric values placed next to arrows represent factor loadings.

Based on the gender- associated issues with the factor structure of the RAS, we conducted a gender-specific analysis of the individual item responses (Figure 1).

**Figure 2:**
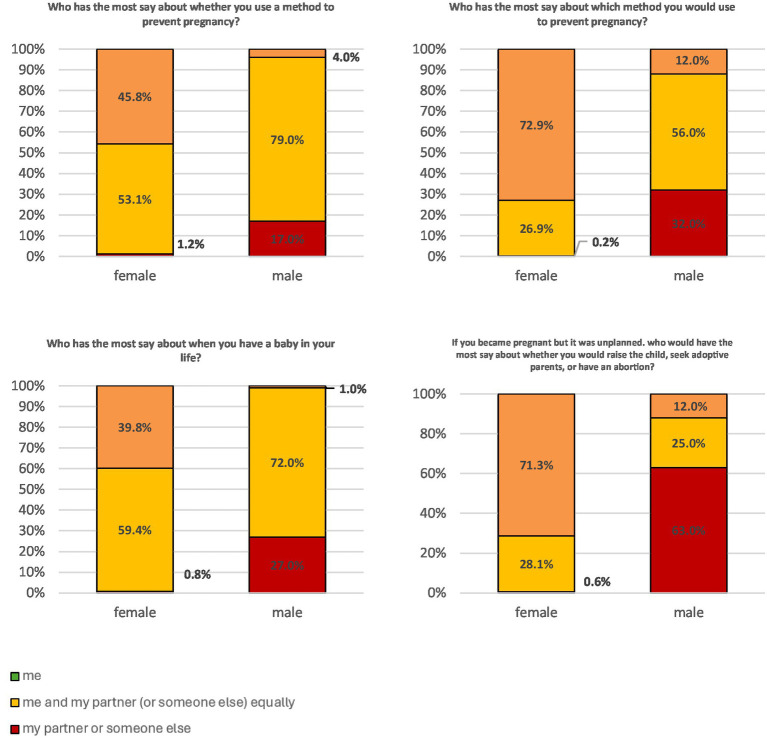
Response patterns for the four items from the ‘decision making’ subscale stratified for male and female participants.

In the domain ‘decision making’ female and male participants reported statistically significant differences on all items (all *p* < 0.001). Female participants reported significantly higher rates of being the sole decision-makers regarding key aspects of reproductive autonomy compared to male participants. This included decisions about the use of contraception (45.8% vs. 4.0%), choice of contraceptive method (72.9% vs. 12.0%), whether to have a child (39.8% vs. 1.0%), and whether to terminate a pregnancy (71.3% vs. 12.0%) (Figure 2).

**Figure 3:**
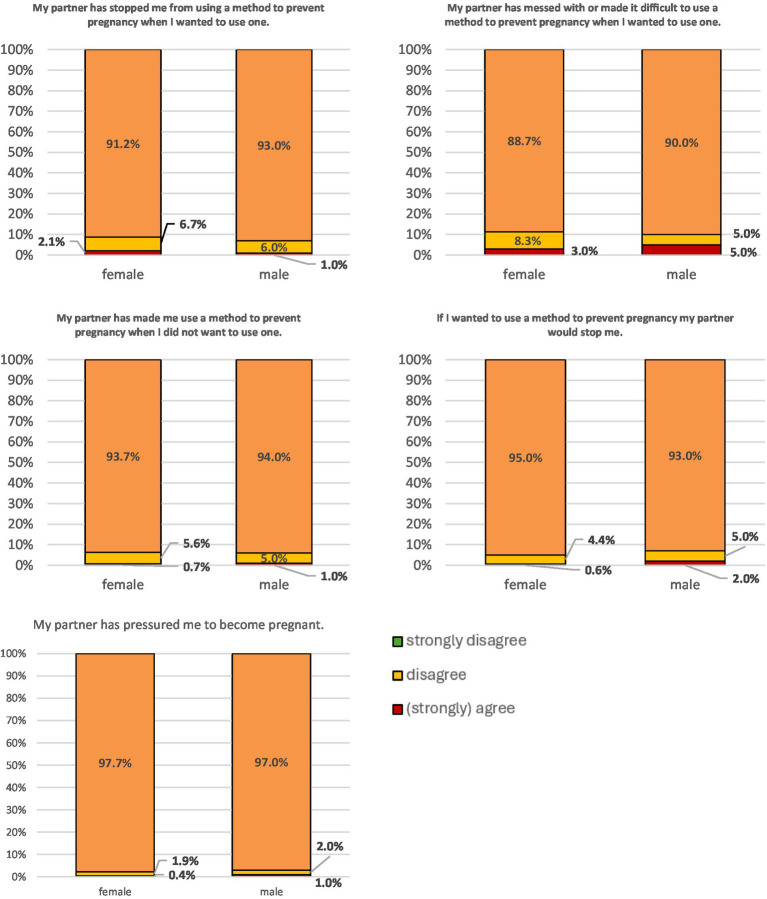
Response patterns for the four items from the ‘freedom of coercion’ subscale stratified for male and female participants.

In terms of the ‘freedom of coercion’, no significant gender difference could be observed for any of the items (*p* = 0.33–0.99). In this context, ‘freedom from coercion’ refers exclusively to the absence of partner-related coercive behaviors as operationalized in the RAS (e.g., pressure to become pregnant, interference with contraceptive use). The scale does not assess institutional, familial, or sociocultural coercion. The vast majority of both male (95–99%) and female (97–99%) participants reported to be free from coercion regarding their reproductive autonomy (Figure 3).

**Figure 4:**
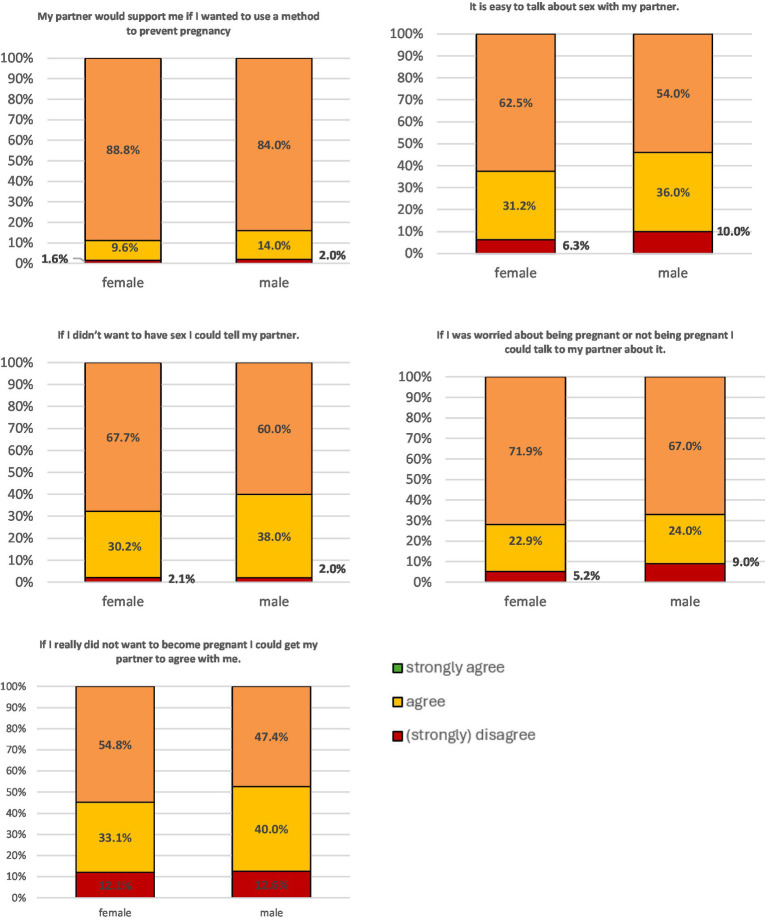
Response patterns for the four items from the ‘communication’ subscale stratified for male and female participants.

In terms of ‘communication’ about reproductive autonomy, no significant gender differences were found any of the items (*p* = 0.07–0.22). Highest disagreement was found with the item “If I really did not want to become pregnant, I could get my partner to agree with me” (female: 12.1%; male: 12.6%) (Figure 4).

### 3.6 Association of RAS scales with sociodemographic factors

In our sample age was positively correlated with higher freedom from coercion (*r* = 0.09, *p* = 0.019), while no significant association was found with decision- making (*p* = 0.67) or communication (*p* = 0.98). None of the subscales showed a significant association with the participants study subject (*p* = 0.27–0.44), religious beliefs (*p* = 0.23–0.50) or highest education of parent (*p* = 0.09–0.97). However, in terms of their sexual orientation participants reported statistically significant mean differences regarding freedom of coercion (*F* = 12.020, *p* < 0.001) and decision making (*F* = 5.362, *p* = 0.005), while no significant difference was found for communication. Post-hoc tests showed that participants with other sexual orientations (which included categories such as ‘pansexual’, ‘asexual’, or ‘undefined’) reported significantly lower mean values for the freedom of coercion subscale than heterosexual (p < 0.001) or homo/bi-sexual participants (p < 0.001), while no significant difference was found between the latter groups (*p* > 0.99). In the decision- making subscale homo−/bi-sexual participants reported higher values than heterosexual participants (*p* = 0.023), while no difference was observed between the other groups (*p* = 0.12–0.99).

## 4 Discussion

The aim of the present study was to validate the RAS in a gender-diverse sample of university students, thereby contributing to the advancement of reproductive autonomy research. Specifically, our objectives were threefold: (1) to translate and psychometrically validate the RAS for use in the German language, (2) to assess its applicability across genders, including men and diverse individuals, and (3) to examine preliminary patterns of reproductive autonomy within a diverse Austrian university student population.

### 4.1 Validation of the German version of the RAS

In the first step, we tested the psychometric properties of the proposed scales. The item-total correlations indicated a satisfactory relationship with the overall construct. However, item 7 (“My partner has made me use a method to prevent pregnancy when I did not want to use one”) had a borderline acceptable correlation of 0.30, suggesting it may not be as strongly connected to the overall scale as other items.

Factor loadings, which show how well each item represents the underlying factor, were generally acceptable, with most items exceeding the predefined threshold of 0.4. This suggests that the items are reasonably associated with their intended factors. Nonetheless, item 14 (‘*If I really did not want to become pregnant, I could get my partner to agree with me*’) had a factor loading of 0.36, which was slightly below the acceptable range, thus indicating it may not optimally align with the assigned factor ‘communication’.

In terms of internal consistency, the ‘decision-making’ and ‘freedom from coercion’ subscales showed acceptable reliability indices, suggesting the items within these subscales consistently measure the same construct. On the other hand, the communication subscales had a slightly lower internal consistency score (*α* = 0.67). Although these scores are just below the commonly accepted threshold of 0.70, they suggest that the items in these subscales may not be as cohesive and that there might be room for improvement. Elimination of item 14 would have resulted in a slightly improved internal consistency of *α* = 0.69, which would still be slightly below the acceptable range. Despite minor psychometric limitations, the Confirmatory Factor Analysis (CFA) supported the original three-factor structure of the RAS, demonstrating an overall good model fit. This means the data fit well with the proposed three-factor model of the RAS. The results suggest that while the RAS generally has acceptable psychometric properties and supports a three-factor structure, certain items (notably items 7 and 14) may need refinement. However, the results of our analysis do not clearly support the decision to exclude those items from the questionnaire. In this instance, we would argue to keep the original form of the questionnaire to avoid confusion with different questionnaire forms across countries and thus allow international comparability of study results.

### 4.2 Assessing the applicability of the RAS across genders

To determine if the RAS is equally usable for male and female participants, we analyzed the gender specific factorial structure of the scale. It should be noted that the RAS was originally developed for use with women and was not specifically designed to be applied to male participants.

The results of this measurement invariance testing highlighted substantial gender-differences in the scale structure. There were significantly more women than men in our cohort, which should be taken into account when interpreting the results. While the model fit well for female participants, the fit was substantially poorer for male participants, especially due to item 10 (“*My partner would support me if I wanted to use a method to prevent pregnancy*”). Removing item 10 from the male sample significantly improved the model fit, indicating that this item might not be appropriate for male participants. The results of the measurement invariance testing were further supported by the gender-specific analyses of the response patterns for each item of the RAS. We found significant differences in the response patterns regarding the ‘decision-making’ subscales for male and female participants. In this subscale, female participants significantly more often reported to be solely responsible for different aspects of contraception. However, given the nature of pregnancy, the answers may carry different meanings for male and female participants. If a female participant reports being solely responsible for reproductive decisions, this indicates a high level of reproductive autonomy. In contrast, a male participant’s response could mean he takes responsibility for contraception, suggesting a progressive view on reproductive autonomy. This result could also be related to generational differences. The participants in this survey were students aged up to 30. A cohort of men over the age of 50 might have produced different results with regard to responsibility for contraception. However, it could also imply that the male wants to control his (female) partner’s reproductive decisions, indicating a patriarchal view that is less aligned with female reproductive autonomy. We would therefore recommend to reversely score the items of the ‘decision-making’ subscale for male participants if the questionnaire is used in a mixed sample. As for the ‘freedom from coercion’ and the ‘communication’ subscales, our results did not indicate a consistent gender difference. However, due to the wording of items 13 (‘*If I was worried about being pregnant or not being pregnant, I could talk to my partner about it*’) and 14 (‘*If I really did not want to become pregnant, I could get my partner to agree with me’*) male participants may find it difficult to respond to these questions.

The RAS was originally developed and validated in a U. S. sample of 1892 women of reproductive age recruited from family planning and abortion clinics. These participants represented a broad range of socioeconomic backgrounds but the recruitment setting likely captured a population already facing challenges in reproductive decision-making (5). Women in the U. S. sample scored lower on average than those in the present study. This discrepancy may be partly explained by differences in the study population: the current sample comprised primarily healthy, young university students in Austria as the cohort was not recruited from a health care setting. Moreover, the relatively comprehensive and accessible Austrian healthcare system may support a higher baseline level of reproductive autonomy compared to the U. S. context.

Subsequent adaptations of the RAS have targeted diverse female populations in varied sociocultural and economic contexts. In Brazil, the scale underwent linguistic simplification to enhance accessibility across educational levels and was modified to include items reflecting financial dependency and fear of violence (9, 10). The Brazilian sample included 140 women, evenly split between rural workers and descendants of Afro-Brazilian slaves—many of whom had limited formal education (10). When adjusted to match the present study’s scale, decision-making scores were comparable, but scores for freedom from coercion and communication were notably lower in the Brazilian context. Importantly, reproductive autonomy in the Brazilian sample was significantly associated with contraceptive use—a relationship not observed in the Vietnamese validation study (7), raising questions about cross-cultural validity and the influence of contextual norms.

In the United Kingdom, the RAS was analyzed in women of reproductive age who had engaged in heterosexual vaginal intercourse within the preceding 12 months. Mean scores are like the Austrian findings at hand (11). These parallels likely reflect comparable legal protections, gender equality measures, and access to reproductive healthcare in Austria and the UK, which may foster similar levels of reproductive autonomy.

Taken together, these diverse applications of the RAS underscore its sensitivity to context—both in terms of cultural norms and structural conditions. While the scale has demonstrated utility across varied settings, findings highlight the need for careful interpretation of results within each population. The present study contributes to this body of research by applying the RAS to a gender-diverse, highly educated, and relatively privileged Austrian student sample.

The RAS has proven to be a valid and contextually appropriate tool for assessing reproductive autonomy in a German-speaking sample of university students, particularly among women.

Reproductive autonomy should be understood not as a fixed attribute but as a dynamic and context-dependent construct, continuously shaped by an individual’s life stage, relational circumstances, gender norms, and broader sociocultural influences. The RAS offers a momentary snapshot, but a more nuanced understanding would benefit from longitudinal research tracking individuals over the course of their reproductive lives.

From a public health and policy perspective, ensuring reproductive autonomy requires more than measurement. It demands structural support: uncomplicated, affordable access to contraception, comprehensive sexual education, and targeted efforts to promote gender-equitable contraceptive responsibility. This includes promoting awareness of sexual and reproductive rights, addressing inequalities in access based on gender and socioeconomic status, and fostering a culture of shared responsibility through education and public health initiatives (12).

Future research should aim to adapt and validate the RAS more inclusively, ensuring that it captures the diverse realities of reproductive autonomy across sexual orientations, gender identities, and family structures.

While the notion of reproductive autonomy in men may appear less urgent given the relative absence of structural reproductive restrictions, meaningful autonomy—especially regarding shared decision-making, sterilization, and participation in abortion-related decisions—deserves further conceptual and empirical exploration (13, 14).

The strength of the RAS lies in its multidimensional approach, capturing decision-making, communication, and freedom from coercion—key components of reproductive autonomy—without being limited to marital status or household structure (5, 13, 15–17).

Further, one major strength of the study is the size of sample and the analysis of persons not in a hospital setting but in an every-day setting. It can reasonably be assumed that this group is gynecologically healthy and thus offers a meaningful contrast to previously studied populations recruited from fertility and abortion clinics, which may be characterized by acute reproductive health needs.

Several limitations should be acknowledged in interpreting the findings of this study. First, the sample comprised university students from Austria, representing a relatively homogenous and highly educated cohort in means of education and age that does not reflect the broader demographic or sociocultural cross-section of the general population.

Second, the sample exhibited a significant gender imbalance, with 520 women and only 100 men completing the survey. This uneven distribution may reflect a higher level of interest in reproductive health topics among women, which is consistent with prior research on gender differences in participation in reproductive health studies (18). While the inclusion of male participants represents an important and novel aspect of this study, it should be noted that the RAS was originally developed for use with women. As discussed, several items may require conceptual and linguistic adaptation —such as rephrasing or reversing items within the decision-making subscale— to ensure relevance and validity when applied to men and gender-diverse individuals.

Third, this application must be considered exploratory. Certain items—particularly those referencing pregnancy, abortion, or partner dynamics—are framed within hetero- and cis-normative contexts, which may limit their applicability to individuals in non-heterosexual or gender-diverse relationships. The current version of the RAS does not account for reproductive scenarios such as adoption, medical assisted reproduction, surrogacy, or broader family planning experiences common in LGBTQ+ communities. As a result, the data primarily reflect the perspectives and experiences of cisgender women and men in presumably heterosexual contexts.

## 5 Conclusion

The RAS offers a valuable and validated framework for assessing reproductive autonomy in German and is well- suited for use among girls and women in European contexts. The present study demonstrates its applicability in a student population, where relatively high levels of reproductive autonomy were observed, varying by age and sexual orientation. However, findings also underscore that women continue to bear disproportionate responsibility for contraception, highlighting persistent gendered dynamics in reproductive decision-making. To promote shared responsibility, comprehensive sexual education and awareness of reproductive rights remain essential. Its current structure limits applicability in male and diverse populations, requiring thoughtful adaptations to ensure relevance across diverse gender identities. To fully realize its potential, future iterations of the RAS must broaden its inclusivity, enhance its cultural and gender sensitivity, and reflect evolving understandings of sexual and reproductive health.

## Data Availability

The raw data supporting the conclusions of this article will be made available by the authors, without undue reservation.
